# Rintatolimod (Ampligen^®^) Enhances Numbers of Peripheral B Cells and Is Associated with Longer Survival in Patients with Locally Advanced and Metastasized Pancreatic Cancer Pre-Treated with FOLFIRINOX: A Single-Center Named Patient Program

**DOI:** 10.3390/cancers14061377

**Published:** 2022-03-08

**Authors:** Hassana el Haddaoui, Rianne Brood, Diba Latifi, Astrid A. Oostvogels, Yarne Klaver, Miranda Moskie, Dana A. Mustafa, Reno Debets, Casper H. J. van Eijck

**Affiliations:** 1 Department of Surgery, Erasmus University Medical Center, 3015 GD Rotterdam, The Netherlands; h.elhaddaoui@erasmusmc.nl (H.e.H.); riannebrood@gmail.com (R.B.); dibalatifi@hotmail.com (D.L.); m.moskie@erasmusmc.nl (M.M.); 2 Laboratory of Tumor Immunology, Department of Medical Oncology, Erasmus University Medical Center, 3015 GD Rotterdam, The Netherlands; a.oostvogels@erasmusmc.nl (A.A.O.); y.klaver@erasmusmc.nl (Y.K.); j.debets@erasmusmc.nl (R.D.); 3 Department of Pathology, The Tumor Immuno-Pathology Laboratory, Erasmus University Medical Center, 3015 GD Rotterdam, The Netherlands; d.mustafa@erasmusmc.nl

**Keywords:** toll-like receptor 3, rintatolimod, immunotherapy, maintenance therapy, pancreatic cancer

## Abstract

**Simple Summary:**

Survival of patients with locally advanced and metastatic pancreatic cancer remains poor after standard care with FOLFIRINOX. It is not known whether treatment with rintatolimod, a TLR-3 agonist, would improve progression-free survival. We investigated the immunomodulatory effects of rintatolimod and its benefits regarding progression-free and overall survival in patients who had been pre-treated with FOLFIRINOX. In long-term survivors (11 patients), we observed that rintatolimod treatment resulted in a lowering of the systemic immune-inflammation index (SIII) as well as the neutrophil to lymphocyte ratio (NLR), whereas peripheral B-cell counts were higher, when compared to short-term survivors (16 patients). Of note, rintatolimod treatment was associated with a higher progression-free and overall survival when compared to matched controls. These results support the effectiveness of adding rintatolimod as a maintenance therapy after FOLFIRINOX treatment in patients with advanced pancreatic cancer. A randomized controlled trial to further determine the efficacy of the drug is recommended.

**Abstract:**

Background: Treatment with the TLR-3 agonist rintatolimod may improve pancreatic cancer patients’ survival via immunomodulation, but the effect is unproven. Methods: In this single-center named patient program, patients with locally advanced pancreatic cancer (LAPC) or metastatic disease were treated with rintatolimod (six weeks total, twice per week, with a maximum of 400 mg per infusion). The primary endpoints were the systemic immune-inflammation index (SIII), the neutrophil to lymphocyte ratio (NLR), and the absolute counts of 18 different populations of circulating immune cells as measured by flow cytometry. Secondary endpoints were progression-free survival (PFS) and overall survival (OS). Subgroup analyses were performed in long-term survivors (>1-year overall survival after starting rintatolimod) and compared to short-term survivors (≤1 year). Results: Between January 2017 and February 2019, twenty-seven patients with stable LAPC or metastatic disease were pre-treated with FOLFIRINOX and treated with rintatolimod. Rintatolimod treatment was well-tolerated. The SIII and NLR values were significantly lower in the 11 long-term survivors, versus 16 short-term survivors. The numbers of B-cells were significantly increased in long-term survivors. Numbers of T cells and myeloid cells were not significantly increased after treatment with rintatolimod. Median PFS was 13 months with rintatolimod, versus 8.6 months in a subset of matched controls (*n* = 27, hazard ratio = 0.52, 95% CI = 0.28–0.90, *p* = 0.007). The median OS was 19 months with rintatolimod, versus 12.5 months in the matched control (hazard ratio = 0.51, 95% CI = 0.28–0.90, *p* = 0.016). Conclusions: Treatment with rintatolimod showed a favorable effect on the numbers of peripheral B cells in patients with pancreatic cancer and improved survival in pancreatic cancer, but additional evidence is required.

## 1. Introduction

The standard therapy for patients with locally advanced pancreatic cancer (LAPC) and metastatic pancreatic cancer is chemotherapy, preferably with FOLFIRINOX or gemcitabine nab-paclitaxel [1,2,3,4]. Survival remains poor, however, as the 2-year survival rate is only 50% for LAPC and 10% for patients with metastatic disease after FOLFIRINOX treatment [1,5]. Pancreatic ductal adenocarcinoma (PDAC) is known to be an immunologically cold tumor [6]. Treatment efficacy is hampered by epigenetic and genetic heterogeneity, stromal desmoplasia, and the infiltration of immunosuppressive cells and dysfunctional CD8+ T cells [6,7,8,9]. Higher levels of dysfunctional CD8+ T cells are present in the tumor, especially in advanced disease stages, when compared to resected PDAC [10]. Treatment of pancreatic cancer might be improved by enhancing the immunogenic response.

A possible novel therapeutic agent for patients with PDAC is rintatolimod, which is a double-stranded RNA (dsRNA) molecule derived from Poly I:C, a synthetic form of viral double-stranded RNA, that activates the toll-like receptor 3 (TLR-3) present on dendritic cells, B-cells, natural killer cells, and pancreatic epithelial cells [11,12,13,14]. Toll-like receptors (TLRs) belong to the family of pattern recognition receptors (PRRs) operating in innate immunity [15]. TLR-3 stimulation potentially increases cytotoxic T-cell activity in PDAC through driving the maturation of dendritic cells and the cross-priming of antigens, as well as inducing the production of anti-tumoral type I interferon that induces the proliferation of cytotoxic T cells [16,17,18,19]. In addition to the immunomodulatory effects, TLR-3 stimulation is theorized to restrain tumor cell proliferation and induce apoptosis in tumor cells [20,21,22]. The immunomodulating effect of rintatolimod has been evaluated in patients with chronic fatigue syndrome (CFS) and rintatolimod treatment was proven safe in various phase II-III trials [23,24,25]. The efficacy of rintatolimod in malignancies is currently being tested in clinical trials [26,27] but has never been tested in pancreatic cancer.

Considering that the systemic immune cell response could be positively affected by treatment with rintatolimod, we investigated the immunomodulatory effects of rintatolimod in pancreatic cancer patients by analyzing systemic inflammation parameters [28,29] and immune cells in the peripheral blood. In addition, we investigated whether rintatolimod improves progression-free survival and overall survival, by comparing the survival rates after treatment with rintatolimod with the survival rates in matched controls.

## 2. Materials and Methods

### 2.1. Patients and Study Design

This prospective single center named patient program was performed at the Erasmus University Medical Center in Rotterdam between January 2017 and February 2019, which means that follow-up data for a minimum of 2 years were available for all included patients. Rintatolimod was provided for compassionate use on a named patient basis, and primary objectives were analyzed as part of the ‘Immune Monitoring in Pancreatic cancer’ study (MEC-2016-575). All patients provided written informed consent before inclusion. A matched control group was selected from a historical cohort that included pancreatic cancer patients who had received FOLFIRINOX between 2012 and 2018. Patients were matched based on age, sex, disease stage, and the number of cycles of FOLFIRINOX. Patients in the historical cohort were matched in a 2:1 ratio to patients who received rintatolimod. Moreover, data from a subset of the matched control group (matched in a 1:1 ratio) with longer progression free intervals after treatment with FOLFIRINOX were used for additional analysis (see definitions). This analysis served to avoid overestimating the effect of rintatolimod on progression-free and overall survival rates.

Inclusion criteria for the study group were LAPC; metastatic PDAC or recurrence after resection; completion of standard of care treatment (i.e., resection and/or chemotherapy); and adequate hematologic, renal, and hepatic function. Exclusion criteria were a history of another malignancy in the previous five years before inclusion and concomitant immunosuppressive medication use. Included in the analysis were only data of patients pretreated with FOLFIRINOX and with radiologically stable disease according to response evaluation criteria in solid tumors (RECIST) criteria 1.1 at least 6 weeks or more after the last cycle before treatment with rintatolimod.

### 2.2. Definitions of Patient Subgroups According to Survival

Patients were divided into two subgroups based on the length of survival after the start of rintatolimod treatment: long-term survivors (overall survival > 1 year) and short-term survivors (overall survival ≤ 1 year). The disease stage was categorized as either LAPC, metastasized disease, or local recurrence after surgery. To adjust for the short progression-free survival interval after the last FOLFIRINOX dose in the matched control group, a subset of matched controls was created that included only patients with a time to progression calculated from the last FOLFIRINOX dose longer than the median time to progression calculated from the first FOLFIRINOX dose.

### 2.3. Treatment Specifics

Patients received treatment for a maximum of three cycles of six weeks or until disease progression. A treatment cycle consisted of twice per week intravenous administration of 200 milligrams for the first two weeks and 400 milligrams for the last four weeks, as recommended by the manufacturer (AIM ImmunoTech, Ocala, FL, USA). Laboratory assessments and peripheral blood sample collections for immunology flow cytometry analysis were obtained at baseline and 5 days after the last infusion of the first cycle. Treatment-related adverse events were reported and graded according to the Common Terminology Criteria for Adverse Events version 5.0 [30]. Progression of disease was assessed by CT imaging scans according to RECIST criteria 1.1 and serum carbohydrate antigen 19-9 examinations every three months during follow-up, if clinically indicated, or when recurrence was suspected.

### 2.4. Endpoints of Study

The primary endpoint was the systemic immune cell response defined as changes in the systemic immune-inflammation index (SIII) [28] and the neutrophil to lymphocyte ratio (NLR) [29], as well as changes in circulating immune cells determined with flow cytometry analysis. The SIII was calculated with the formula: (neutrophils × platelets)/lymphocytes and the NLR with the formula: neutrophils/lymphocytes. The secondary outcomes were progression-free survival (PFS), calculated from the start of FOLFIRINOX until progression according to RECIST criteria 1.1, and overall survival (OS), calculated from the start of FOLFIRINOX until death.

### 2.5. Flow Cytometry Analysis

Blood samples were collected in 10 mL of EDTA blood collection tubes and immune cells were quantified within a maximum of 24 h after blood draw. Flow cytometry was performed to determine the granulocyte, monocyte, and lymphocyte subset cell counts in the circulating blood of patients treated with rintatolimod (Table 1), in the manner reported previously [31]. For financial reasons, only the peripheral blood samples of 18 out of 27 patients were analyzed. Blood was stained with monoclonal antibodies and analyzed using the BD FACSCelesta™ flow cytometer (Becton Dickinson, Franklin Lakes, NJ, USA). Absolute immune cell counts were determined using fluorescent microspheres (Beckman Coulter, Brea, CA, USA). The monoclonal antibody panel has been described previously [31], and has been optimized and compensated using fluorescence minus one controls. Data were analyzed using FlowJo software (Tree Star, San Carlos, CA, USA).

### 2.6. Statistical Analysis

Paired measurements were analyzed using the paired sampled *t*-test or Wilcoxon-signed rank test, according to the distribution of the data. For comparison of subgroups, independent *t*-tests or Mann–Whitney U tests were performed, according to the distribution of the data. Progression-free and overall survival were presented as Kaplan–Meier curves and compared using log-rank tests. Hazard ratios were calculated using Cox proportional hazards models. All tests were two-sided and performed at the *p* = 0.05 significance level. All statistical analyses were performed with SPSS version 28.0.1.

## 3. Results

### 3.1. Patient Treatments

**Figure 1:**
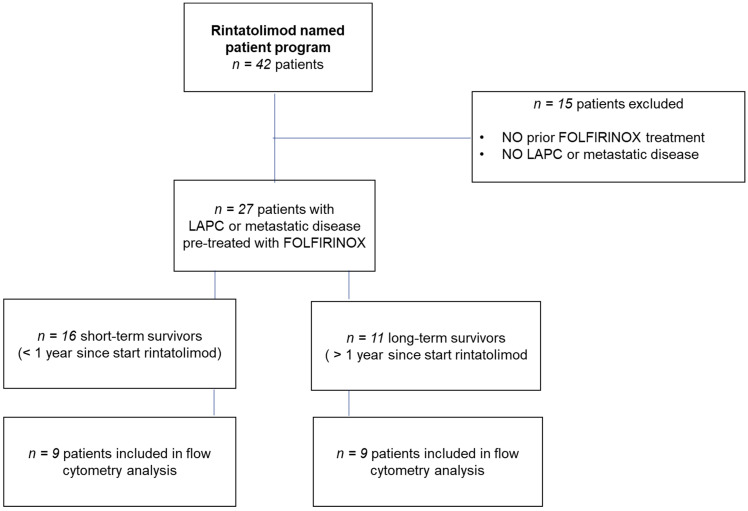
Profile of the single-center name patient program.

From January 2017 to February 2019 a total of forty-two patients with LAPC or metastasized disease were treated with rintatolimod (Figure 1). Twenty-seven of those had been treated with FOLFIRINOX prior to rintatolimod. Only 25 patients of them completed the first cycle of rintatolimod. Patient characteristics are presented in Table 2. The baseline characteristics—age, sex, disease stage, and number of FOLFIRINOX cycles—were not statistically significantly different between the rintatolimod treatment group and the matched controls. Based on OS after rintatolimod treatment, we distinguished 11 long-term survivors and 16 short-term survivors.

### 3.2. Rintatolimod Was Well-Tolerated

Serious adverse events (Common Terminology Criteria for Adverse Events Version 5.0 grade 3–5) after treatment with rintatolimod were not observed (Table 3). Treatment with rintatolimod was well-tolerated. The majority of the patients reported treatment related chills (shiver) or fatigue and muscle pain (myalgia). Other reported adverse events were facial flushing and dyspepsia. Two patients had a grade 1–2 allergic reaction.

### 3.3. Rintatolimod Changes Indexes of Immune Effector Cells in the Circulation

**Figure 2:**
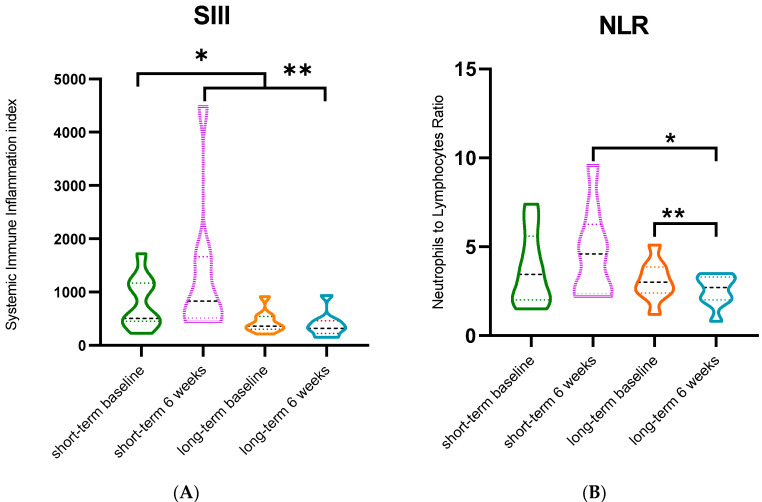
(**A**) Systemic immune inflammation index (SIII) in short-term survivors (*n* = 9) and long-term survivors (*n* = 9). *: *p* = 0.010 (Mann–Whitney U test); **: *p* = 0.0005 (Mann–Whitney U test_. (**B**) Neutrophil to lymphocyte ratio (NLR) in short-term survivors and long term-survivors. *: *p* = 0.006 (Mann–Whitney U test); **: *p* = 0.014 (Mann–Whitney U test).

To determine the systemic effect of rintatolimod, we analyzed SIII and NLR values and numbers of circulating immune cells at the baseline and after six weeks of rintatolimod treatment. We did not find a significant decrease of SIII values in all 27 patients. The median SIII was 562.2 at baseline, versus 538.0 after six weeks (*p* = 1.000). The analysis based on OS showed significantly decreased SIII values in the long-term survivors at both blood sampling timepoints (Figure 2A). The median SIII at the baseline was 359.2, versus 319.6 after six weeks in the long-term survivors, (*p* = 0.003), while in the short-term survivors, the SIII increased (median 948.0 versus 1142.0, (*p* = 0.104)). Moreover, we also found higher SIII values at the baseline and after six weeks in the short-term survivors in comparison to the long-term survivors. (Figure 2A). Median SIII at baseline in short- and long-term survivors were 948.0, versus 359.2, respectively (*p* = 0.010). Median SIII after six weeks in short-term survivors was 1142.0, versus 319.0 in long-term survivors (*p* = 0.005). We did not find a significant decrease of NLR values in all 27 patients. The median NLR at baseline was 3.5 versus 3.0 after six weeks (*p* = 0.770). NLR was also analyzed based on OS groups, as shown in Figure 2B. The NLR after six weeks of treatment in the short-term survivors was significantly higher than that in the long-term survivors, with medians of 4.8 and 2.7, respectively (*p* = 0.006). Furthermore, in the long-term survivors, the NLR after treatment was significantly lower than that at baseline, with medians of 3.0 and 2.7, respectively (*p* = 0.014). The decrease in NLR in the short-term survivors was not significant however, with medians of 4.7 and 4.8, respectively (*p* = 0.268).

### 3.4. Rintatolimod Enhances Numbers of B Cells in the Circulation

**Figure 3:**
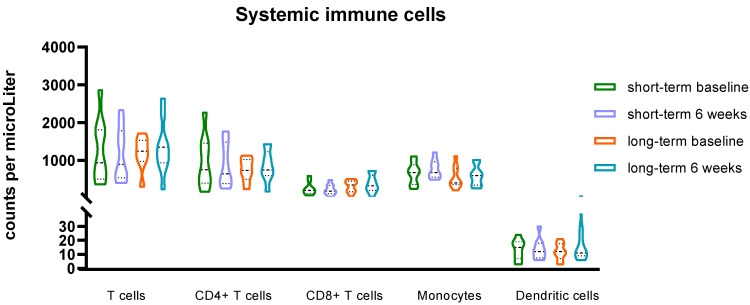
Systemic immune cells in short-term survivors (*n* = 9) versus long-term survivors (*n* = 9). All *p*-values ≥ 0.05 using a paired sample *t*-test.

**Figure 4:**
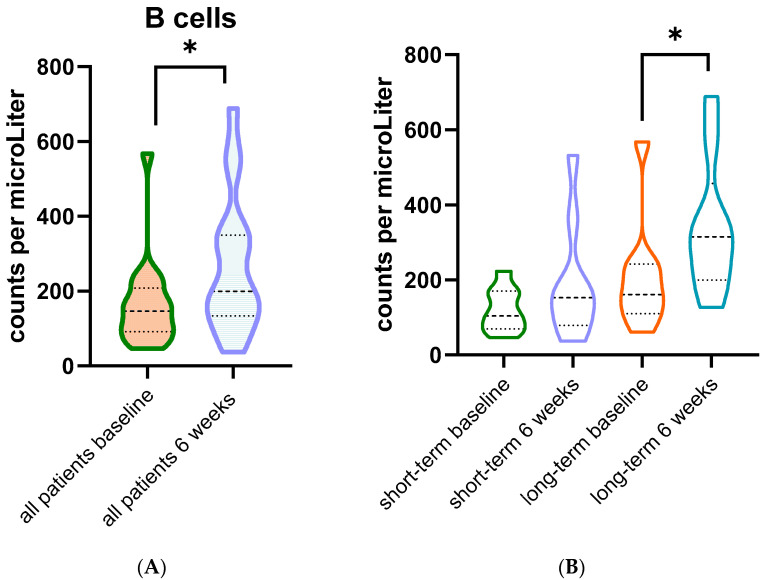
(**A**) B cells in circulating blood of all patients treated with rintatolimod (*n* = 18, *: *p* = 0.001 from paired sample *t*-test). (**B**) B cells in circulating blood of short-term survivors (*n* = 9) and long-term survivors (*n* = 9 *: *p* = 0.002 from paired sample *t*-test).

Quantification of immune cell populations revealed a significant increase only in circulating B cells (i.e., CD3-, CD19+ cells) after six weeks of treatment in the 18 samples measured with flow cytometry (Figure 3 and Figure 4). Numbers of T cells and myeloid cells were not significantly increased after six weeks of treatment (all *p*-values > 0.05) (Figure 3). The mean difference (±standard deviation) in B-cell counts after six weeks was 102.5 (±107.2)/uL in all patients (*p* = 0.001). Subgroup analysis in short-term survivors (*n* = 9) and long-term survivors (*n* = 9) showed increased B-cell counts after treatment in the long-term survivors with a mean difference of 133.9 (±92.1) (*p* = 0.002).

### 3.5. Rintatolimod Treatment Associate with Extended Progression-Free and Overall Survival

All groups (rintatolimod treatment group (*n* = 27), matched controls (*n* = 54) and subset of matched controls (*n* = 27)) were comparable with regard to age, sex, disease stage, and number of FOLFIRINOX cycles (Table 2). Most of the patients in all groups were males in the metastatic disease stage. The median number of FOLFIRINOX cycles was 8.0. The total matched control group was incomparable to the rintatolimod group in terms of progression-free intervals before the start of treatment with rintatolimod (Table 2). The matched controls had a significantly shorter median progression-free interval calculated from the last FOLFIRINOX dose compared to the time in months from last FOLFIRINOX dose to start of rintatolimod in the treatment group. The progression-free interval was comparable between both groups.

**Figure 5:**
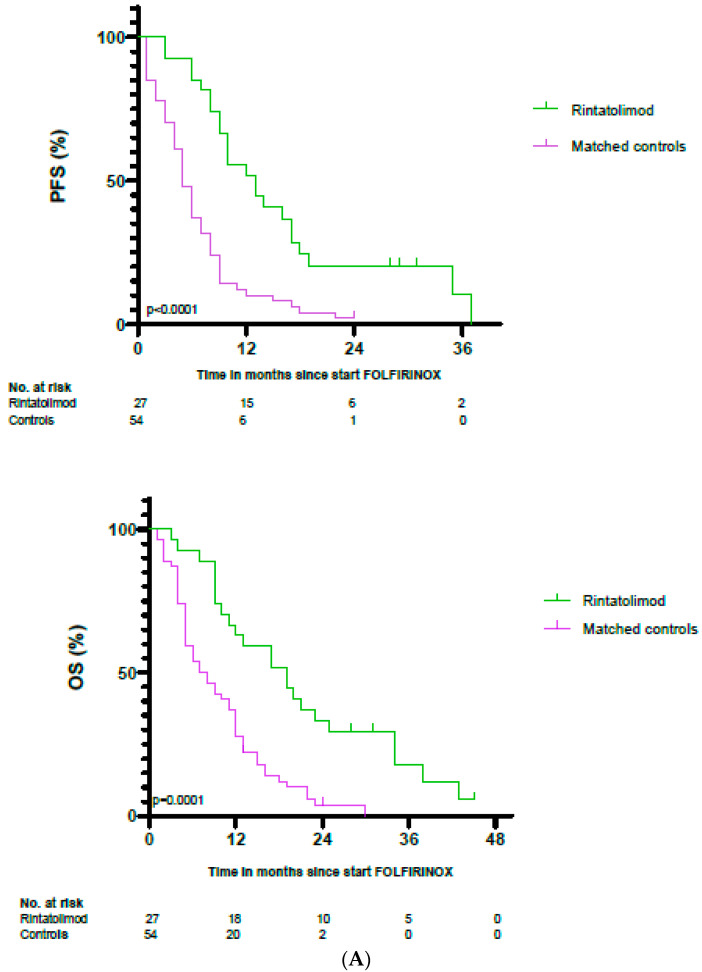

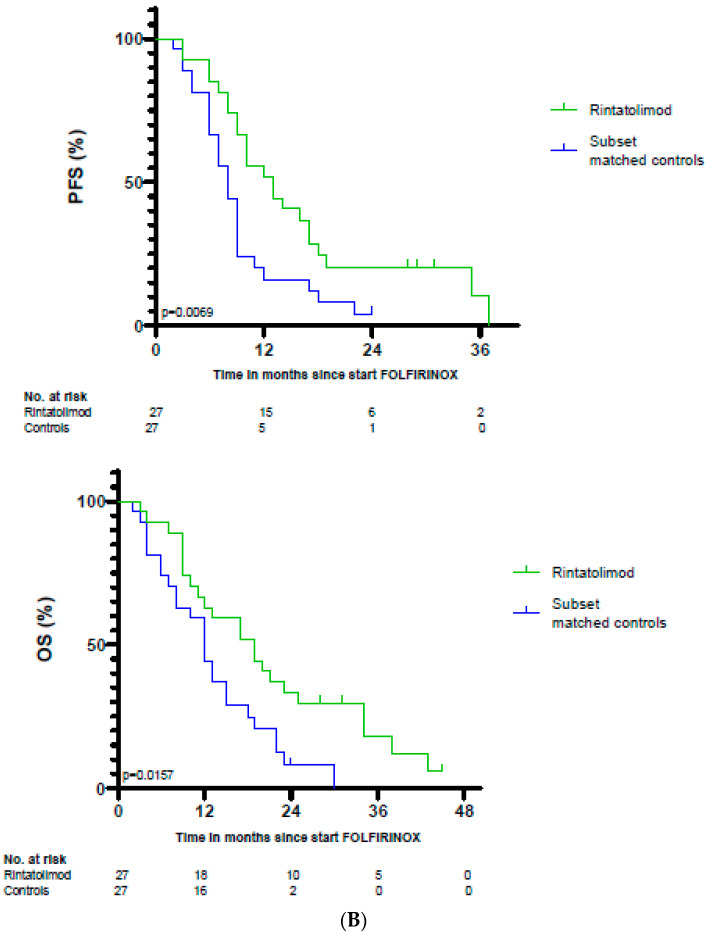
(**A**) Kaplan–Meier estimates for progression-free survival and overall survival of patients with locally advanced and metastatic pancreatic cancer treated with rintatolimod (*n* = 27) compared to matched controls (*n* = 54). (**B**) Kaplan–Meier estimates for progression-free survival and overall survival of patients with locally advanced and metastatic pancreatic cancer treated with rintatolimod (*n* = 27) compared to subset of matched controls (*n* = 27). *p*-values are from the Cox proportional hazards model.

The PFS of patients who received rintatolimod was significantly longer (13 months) than that of both matched controls and the subset of matched controls (5 and 8.6 months, respectively) (Figure 5A). The hazard ratio for progression in the treatment group compared to the subset of matched controls was 0.51 (95% CI = 0.28–0.90, *p* = 0.007). The OS of patients who received rintatolimod was significantly longer (19 months) than that of both matched controls and the subset of matched controls (7.5 and 12.5, respectively) (Figure 5B). The hazard ratio for death in the treatment group was 0.52 (95% = CI 0.28–0.90, *p* = 0.016). None of the patients were lost before follow-up. The censoring in the Kaplan– Meier plots can be ascribed to patients who were still alive at the time of analysis. Notably, at the time of analysis in November 2021, three patients with metastasized disease were still alive.

## 4. Discussion

This is the first study investigating the immunomodulatory effect of the TLR-3 agonist rintatolimod in patients with locally advanced or metastatic pancreatic cancer following FOLFIRINOX therapy. We hypothesized that treatment with rintatolimod boosts the anti-tumoral immunity. For this reason, we investigated whether treatment with rintatolimod controls tumor progression and thus lengthens the progression-free interval after standard care with FOLFIRINOX. We found lower SIII values at the baseline and both lower SIII and NLR values after six weeks of treatment with rintatolimod in long-term survivors compared to short-term survivors. The finding of a significantly lower SIII, after rintatolimod treatment in the long-term survivors, is in line with that of a previous study, in which a SIII higher than 900 was independently associated with poor prognosis in resectable pancreatic cancer patients [28]. Furthermore, a recently published study found a strong association between the NLR and prognosis in pancreatic patients with locally advanced and metastatic disease [29]. Moreover, we found increased B-cell counts in the circulating blood after treatment, which concurs with the findings of previous studies that evaluated the broad effect of TLR-3 signaling in both the innate and adaptive immune response [13,15]. B cells are involved in modulating T-cell and innate immune responses via amongst other things antigen processing and presentation [32]. Furthermore, stimulation of TLRs expressed on B cells regulates various steps in B-cell development and B-cell activation [33], specifically regulation of B-cell cytokine secretion, antigen presentation, Ig isotype switching, and B-cell survival [33]. The role of B cells in pancreatic cancer is diverse and their exact role in tumor progression remains unknown [34,35]. Interestingly, our subgroup analysis showed significantly increased numbers of B cells only in long-term survivors. This finding concurs with that of previous studies evaluating the prognostic value of infiltrating B cells in pancreatic cancer [36,37]. Higher levels of B cells in tertiary lymphoid tissue in the tumor predicted longer survival in retrospective data of 104 PDAC patients [36]. In addition, Brunner et al. reported higher amounts of tumor-infiltrating B cells in long-term survivors, when compared to matched controls [37]. However, the association of higher B cells in the circulation of long-term survivors that we found has not been reported before.

Although it was previously reported that the activation of TLR-3 with rintatolimod enhances the maturation of DCs which could result in more activated (CD4+ and CD8+) T cells that establish anti-tumor immunity [17,38], we did not find significantly increased circulating DC or T-cell counts after six weeks of treatment with rintatolimod. However, DC are very sensitive cells that represent < 0.2–0.1% of the total circulating immune cells in pancreatic cancer patients [39]. To detect them accurately, blood samples should be measured almost immediately after blood sampling and not within 24 h. Therefore, we think that flow cytometry analysis of the total DCs may be inconclusive. In addition, we did not investigate the effective CD4+ and CD8+ T cells that infiltrate the tumor site and tumor-draining lymph nodes.

Importantly, our investigation indicated that patients treated with rintatolimod had a longer median PFS and OS than that of matched control patients who did not receive rintatolimod. At the time of analysis in November 2021, three patients with the metastasized disease were still alive. Notably, these three patients had received a total of three cycles of rintatolimod. The longer survival rates could be the effect of TLR-3 signaling. Although we acknowledge that the exact role of TLR-3 signaling in PDAC is unknown, TLR-3 agonists were found to enhance tumor cell lysis by human γδ T cells in human pancreatic cells. The effect of the agonist was also found to be TLR-3 dependent [40]. This could mean that individual different responses to rintatolimod in this study could be present, depending on the level of TLR-3 expression in the tumor cells.

Our study has some limitations. Most importantly, the small number of selected patients in the study group and the lack of randomization increase the risk of selection bias. The treatment group was more likely to include patients with a favorable prognosis than was the control group. For example, patients that received rintatolimod might be more motivated or in better overall physical condition. To minimize confounding factors, patients in the treatment group were matched to comparable controls. However, the distribution of other variables that might determine prognosis, such as the performance status or the SIII and cancer antigen 19.9 (CA 19-9) at the start of FOLFRINOX, was not available for the matched controls and therefore not accounted for.

Furthermore, although the increase in numbers of B cells was significant after treatment with rintatolimod in all patients, the absence of a significant decrease in SIII and NLR values in the short-term survivors might be ascribed to the pre-existent impaired recovery of the immune system after completion of chemotherapy. Chemotherapy is known to alter immune responses, and research in breast cancer patients suggests that the recovery of the immune system may occur not until up to twelve months after completion [41,42]. Since rintatolimod is given after FOLFIRINOX, the higher immune cell counts during treatment with rintatolimod may reflect the natural recovery of the immune system.

In conclusion, this is the first named patient program study that describes the use of rintatolimod as a novel therapy to maintain stable advanced pancreatic cancer in patients who were previously treated with FOLOFIRINOX. Measures of peripheral immune response were favorable in patients who survived more than 12 months, but not those who survived less than 12 months following start of rintatolimod treatment. Moreover, treatment with rintatolimod was well-tolerated and associated with a higher PFS and OS compared to matched controls. We propose that rintatolimod could potentially be an effective maintenance therapy after systemic chemotherapy for patients with advanced pancreatic cancer. A randomized controlled trial should be performed to further determine the efficacy of the drug, and to identify patients who would maximally benefit from this new therapy.

## Figures and Tables

**Table 1:** Markers Used in Flow Cytometry to Identify Immune Cells in Peripheral Blood.

	Cell Type	Marker
Granulocytes	Eosinophils	CD15+, CD16-
	Mature neutrophils	CD15^high^, CD16^high^
	Immature neutrophils	CD15+, CD16+
Monocytes	Monocytes Dendritic cells	CD14+, CD16- CD14-, CD16-, CD11c+
Lymphocytes	B cells	CD3-, CD19+
	NK cells	CD3-, CD56+, CD16+/-
	T cells	CD3+
	T helper cells	CD3+, CD4+
	Killer T cells	CD3+, CD8+

**Table 2:** Patient characteristics.

	*N* = 27 Rintatolimod Group	*N* = 54 Matched Controls	*N* = 27 Subset Matched Controls	*p*-Value
Age, median (range)	63 (44–73)	62 (44–78)	65 (46–78)	0.684 * [1] 0.563 * [2]
Male, *n* (%) Female, *n* (%)	19 (70.4) 8 (29.6)	38 (70.4) 16 (29.6)	18 (66.7) 9 (33.3)	1.000 ** [1] 0.770 ** [2]
Disease stage *n* (%)				
LAPC	5 (18.5)	10 (18.5)	5 (18.5)	1.000 ** [1] 0.634 ** [2]
Metastatic disease	16 (59.3)	32 (59.3)	13 (48.2)	
Tumor recurrence after surgery	6 (22.2)	12 (22.2)	9 (33.3)	
FOLFIRINOX cycles, median (range)	8 (1–12)	8 (1–12)	8 (1–12)	0.241 * [1] 0.727 * [2]
Time in months from last FOLFIRINOX dose to start Rintatolimod, median (range)	3.9 (0.4–28)	NA	NA	0.009 Ɨ [1] 0.478 Ɨ [2]
Progression-free interval from last FOLFIRINOX to progression, median months (range)	NA	2.2 (0.0–20.8)	3.4 (2.3–20.8)	

*: *p*-value from independent sample *t*-test; **: *p*-value from chi-square test; Ɨ: *p*-value from Mann–Whitney U test [1] comparing the rintatolimod group and the matched controls [2] comparing the rintatolimod group and a subset of the matched controls.

**Table 3:** Adverse events.

	Total *N* = 27	
	CTCAE* Grade 1–2 *n*(%)	CTCAE Grade 3–5 *n*(%)
Musculoskeletal and connective tissue disorders	9(33.3)	0(0)
Myalgia	8(29.6)	
Back pain	1(3.7)	
General disorders and administration site conditions	22(81.5)	0(0)
Fatigue	8(29.6)	
Chills	14(51.8)	
Vascular disorders	2(7.4)	0(0)
Flushing		
Gastrointestinal disorders	3(11.1)	0(0)
Dyspepsia		
Nervous system disorders	3(11.1)	0(0)
Headache		
Immune system disorders	2(7.4)	0(0)
Allergic reaction		

* Common Terminology Criteria for Adverse Events (CTCAE) version 5.0.

## Data Availability

All data presented in this manuscript are available on request from the corresponding author.
